# Development and validation of a novel nomogram to predict postoperative pancreatic fistula after pancreatoduodenectomy using lasso-logistic regression: an international multi-institutional observational study

**DOI:** 10.1097/JS9.0000000000000695

**Published:** 2023-09-05

**Authors:** Zongting Gu, Yongxing Du, Peng Wang, Xiaohao Zheng, Jin He, Chengfeng Wang, Jianwei Zhang

**Affiliations:** a Department of Hepatobiliary and Pancreatic Surgery and Minimally Invasive Surgery, Zhejiang Provincial People’s Hospital, Affiliated People’s Hospital, Hangzhou Medical College, Hangzhou, Zhejiang; b Department of Pancreatic and Gastric Surgery, National Cancer Center/Cancer Hospital, Chinese Academy of Medical Sciences and Peking Union Medical College, Beijing; c Shanxi Province Cancer Hospital/ Shanxi Hospital Affiliated to Cancer Hospital, Chinese Academy of Medical Sciences/Cancer Hospital Affiliated to Shanxi Medical University, Taiyuan, Shanxi, People’s Republic of China; d Department of Surgery, Johns Hopkins Medical Institutions, Baltimore, Maryland, USA

**Keywords:** deep surgical site infection, delayed gastric emptying, nomogram, pancreaticoduodenectomy, postoperative pancreatic fistula

## Abstract

**Background:**

Existing prediction models for clinically relevant postoperative pancreatic fistula (POPF) after pancreatoduodenectomy (PD) lack discriminatory power or are too complex. This study aimed to develop a simple nomogram that could accurately predict clinically relevant POPF after PD.

**Methods:**

A high-volume, multicenter cohort of patients who underwent PD from the American College of Surgeons-National Surgical Quality Improvement Program database in the United States during 2014–2017 was used as the model training cohort (*n*=3609), and patients who underwent PD from the Pancreatic Center of the National Cancer Center Hospital in China during 2014–2019 were used as the external validation cohort (*n*=1347). The study used lasso penalized regression to screen large-scale variables, then logistic regression was performed to screen the variables and build a model. Finally, a prediction nomogram for clinically relevant POPF was established based on the logistic model, and polynomial equations were extracted. The performance of the nomogram was evaluated by receiver operating characteristic curve, calibration curve, and decision curve analysis.

**Results:**

In the training and validation cohorts, there were 16.7% (601/3609) and 16.6% (224/1347) of patients who developed clinically relevant POPF, respectively. After screening using lasso and logistic regression, only six predictors were independently associated with clinically relevant POPF, including two preoperative indicators (weight and pancreatic duct size), one intraoperative indicator (pancreatic texture), and three postoperative indicators (deep surgical site infection, delayed gastric emptying, and pathology). The prediction of the new nomogram was accurate, with an area under the curve of 0.855 (95% CI: 0.702–0.853) in the external validation cohort, and the predictive performance was superior to three previously proposed POPF risk score models (all *P*<0.001, likelihood ratio test).

**Conclusions:**

A reliable lasso-logistic method was applied to establish a novel nomogram based on six readily available indicators, achieving a sustained, dynamic, and precise POPF prediction for PD patients. With a limited number of variables and easy clinical application, this new model will enable surgeons to proactively predict, identify, and manage pancreatic fistulas to obtain better outcomes from this daunting postoperative complication.

## Introduction

Highlights

- Existing prediction models for CR-POPF after pancreatoduodenectomy lack discriminatory power or are too complex.
- Lasso-logistic regression was used to develop a simple nomogram that could accurately predict CR-POPF.
- Weight, pancreatic duct size, pancreatic texture, deep surgical site infection, delayed gastric emptying, and pathology increased the occurrence of CR-POPF.
- The novel nomogram is based on six readily available indicators, achieving a sustained, dynamic, and precise CR-POPF prediction for pancreatoduodenectomy patients.

Pancreatoduodenectomy (PD) is the mainstay of treatment for periampullary tumors^1^. Despite technical advancements, PD is considered one of the most challenging operations in abdominal surgery due to its complex procedures, lengthy operative times, extensive injuries, and postoperative complications^2^. Common complications of PD include pancreatic fistula (PF), anastomotic leakage, postoperative bleeding, abdominal abscess, and delayed gastric emptying (DGE)^3^. Postoperative pancreatic fistula (POPF) is a serious complication, with an incidence of up to 50%, and is closely associated with other complications, as well as systemic deterioration, such as septic shock, organ failure, and death^4^. Predicting the risk of POPF in advance can optimize individual treatment strategies, such as drainage tube placement and the use of somatostatin analogs. Thus, developing a POPF risk prediction model is crucial for dynamic and accurate POPF risk prediction, guiding patient stratification, and preventive measures to reduce POPF incidence and ultimately improve the prognosis.

Several studies have attempted to address this issue, including the logistic regression-based pancreatic fistula risk score (FRS) system^5^, as well as the subsequent alternative FRS (a-FRS) system^6^ and updated a-FRS (ua-FRS) system^7^. However, due to the limited inclusion of preoperative and intraoperative indicators, the small sample size used to develop these scoring systems, and the lack of large-scale external validation, their discriminative ability and applicability remain unsatisfactory. For instance, in a large validation cohort from India, ua-FRS slightly outperformed FRS and a-FRS in terms of discriminatory ability, but the difference was modest, with area under the receiver operating characteristic curve (AUC) values of 0.70, 0.65, and 0.69, respectively^8^. Consequently, there is still room for optimization of the POPF prediction model^9^. Nomograms, which are also based on logistic regression but include postoperative indicators, have recently been developed^10–12^. Nomograms accurately predicted and discriminated POPF, indicating that the inclusion of postoperative indicators may improve the discriminatory power of the model. However, most reported nomograms are based on smaller cohorts, lack external validation, or contain too many postoperative indicators, especially those related to the diagnosis of POPF, such as drainage fluid amylase^10,12^, which can inflate the discrimination of the model and weaken its usability. Theoretically, all indicators before the establishment of a POPF diagnosis have predictive value. Increasing evidence shows the correlation between postoperative indicators of surgical site infections (SSI) and DGE and the occurrence of POPF^13–15^. However, the predictive value of these indicators for POPF needs to be further clarified.

This study utilized a multicenter high-volume cohort of patients who underwent PD from the ACS-NSQIP database in the United States between 2014 and 2017 to develop a novel nomogram for POPF prediction using reliable lasso-logistic regression. The nomogram incorporated a broad range of perioperative indicators and was externally validated by a cohort from the Pancreatic Center of the National Cancer Center (NCC) Hospital in China between 2014 and 2019, providing a sustained, dynamic, and precise POPF prediction for PD patients.

## Materials and methods

### Patients study cohort

**Figure 1:**
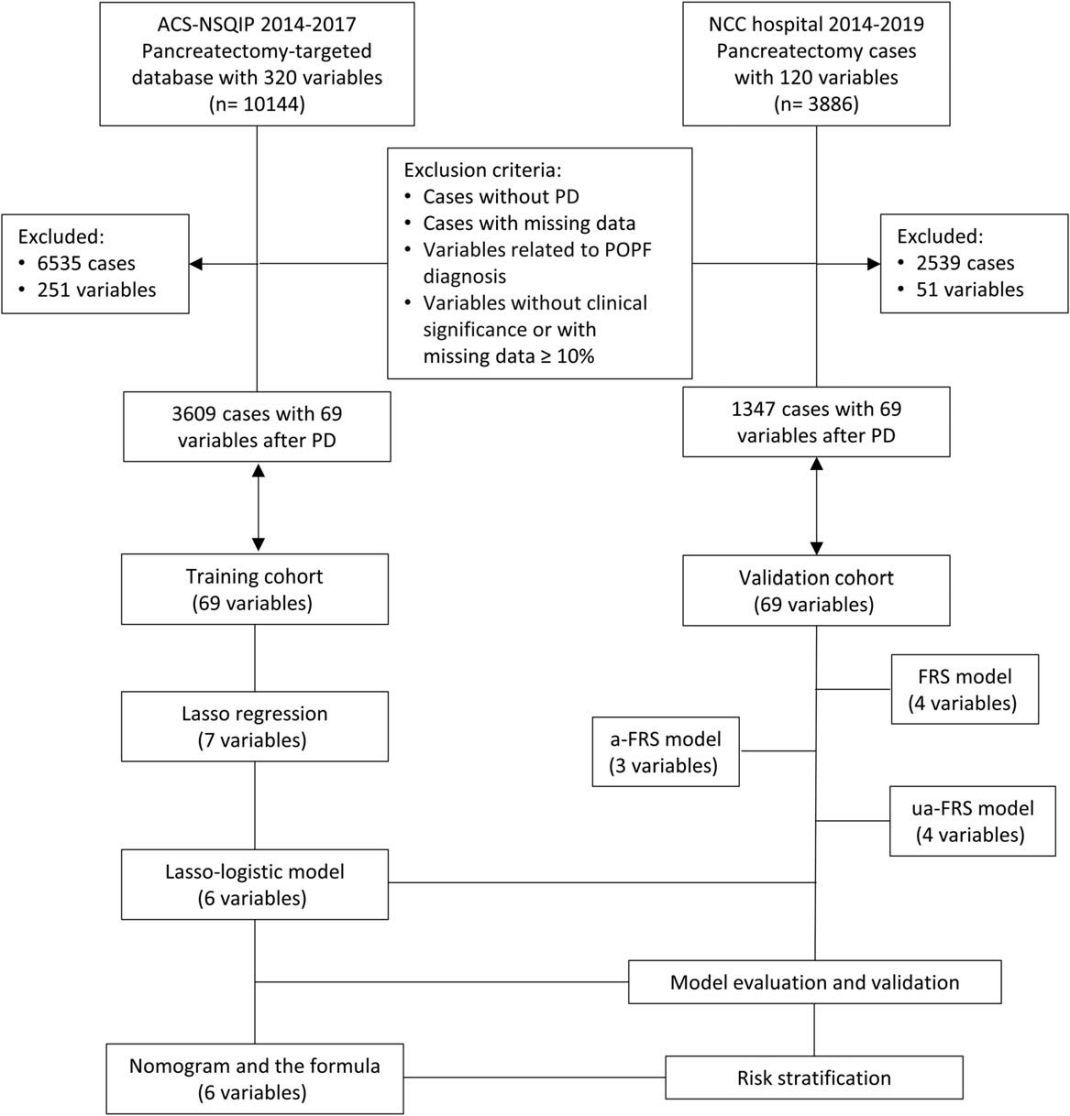
Study flowchart. PD, pancreatoduodenectomy; POPF, postoperative pancreatic fistula. POPF was defined based on the ISGPS 2016 definition.

The training cohort for this study was obtained from 2014 to 2017 pancreatectomy-targeted American College of Surgeons (ACS)-National Surgical Quality Improvement Program (NSQIP) database^16^. The ACS-NSQIP database is a quality improvement database that collects perioperative data within 30 days after surgery from multiple centers across the United States. It is maintained by certified clinical reviewers and subject to quality audits^17^. Patients who underwent PD for pancreatectomy from 2014 to 2017 were identified and included using CPT codes (48 150, 48 152, 48 153, and 48 154). The external validation cohort was obtained from the Pancreatic Center of the NCC Hospital in China during almost the same period (2014–2019). Cases that did not undergo PD and cases with missing data were excluded. Additionally, indicators that directly led to the diagnosis of PF, such as amylase values of postoperative drainage fluid, and indicators without potential clinical significance, such as nursing methods, were also excluded. Figure 1 illustrates the flowchart for data screening and research. The clinical prediction model’s design, validation, and reporting followed the TRIPOD guidelines for multivariable prediction models^18^. As the study exclusively utilized nonidentifiable pre-existing data, it was deemed exempt from the requirement for informed consent procedures in accordance with the Declaration of Helsinki and the Ethical Guidelines for Clinical Studies (No. P-2021-1230). This study was approved by the ethics committee of the Chinese NCC Hospital (No. 17-168/1424). This retrospective study was registered with ResearchRegistry.com (Unique Identification Number: researchregistry8530). Data has been reported in line with strengthening the reporting of cohort, cross-sectional, and case–control studies in surgery (STROCSS) 2021 criteria^19^ (Supplemental Digital Content 1, http://links.lww.com/JS9/A951).

### Definitions and indicators

According to the 2016 ISGPS definition^20^, the severity of POPF is classified as grade A, grade B, and grade C. Grade A, also known as a biochemical leak, is not considered clinically significant, whereas grade B/C POPF is considered clinically relevant (CR-POPF). DGE is defined according to the 2007 ISGPS definition^21^. The definitions of POPF and DGE in ACS-NSQIP are consistent with ISGPF guidelines (https://www.facs.org/-/media/files/quality-programs/nsqip/pt_nsqip_puf_userguide_2017.ashx). The diagnosis of grade B/C CR-POPF is typically established when complications such as abdominal fluid infection or DGE occur and require clinical intervention or reoperation^22^. SSI is defined according to the guidelines provided by the Centers for Disease Control and Prevention (CDC) in the United States^23^. Based on the depth of the SSI, SSI is classified into three types: superficial incisional SSI, deep incisional SSI, and organ or space SSI. For the purposes of this study, deep incisional SSI and organ or space SSI have been combined into a single category known as deep SSI. A pancreatic duct diameter greater than 3 mm is generally considered abnormal and may indicate pathological conditions^24^. Therefore, in the ACS-NSQIP database, the pancreatic duct diameter was grouped using 3 mm as the cutoff value. The study cohort included a total of 69 perioperative indicators, comprising 38 preoperative indicators, 5 intraoperative indicators, and 26 postoperative indicators. Table 1 provides specific information on each indicator. Notably, for practical applicability of the prediction model, all postoperative indicators related to the diagnosis of POPF were excluded (Fig. 1). Additionally, the predictors for each of the three models of FRS^5^, a-FRS^6^, and ua-FRS^7^ were determined according to the corresponding literature.

**Table 1:** Baseline characteristics of study cohorts.

	Perioperative variables	Training cohort	Validation cohort
	Total	3609	1347
Preoperative parameters	Age, mean (SD), years	64.8 (11.4)	65.6 (11.4)
	Sex (%)		
	Female	1651 (45.7)	599 (44.5)
	Male	1958 (54.3)	748 (55.5)
	Height (cm), mean (SD), cm	168.3 (10.5)	167.9 (10.7)
	Weight (kg), mean (SD), kg	77.8 (19.2)	77.5 (18.6)
	BMI, mean (SD), kg/m2	27.4 (5.9)	27.4 (5.6)
	Diabetes (%)		
	None	2665 (73.8)	980 (72.8)
	Noninsulin dependent	492 (13.6)	183 (13.6)
	Insulin dependent	452 (12.5)	184 (13.7)
	Smoker (within one year) (%)		
	No	2932 (81.2)	1104 (82)
	Yes	677 (18.8)	243 (18)
	Dyspnea (%)		
	No	3400 (94.2)	1270 (94.3)
	Moderate/rest	209 (5.8)	77 (5.7)
	History of severe COPD (%)		
	No	3461 (95.9)	1291 (95.8)
	Yes	148 (4.1)	56 (4.2)
	Ascites (%)		
	No	3595 (99.6)	1343 (99.7)
	Yes	14 (0.4)	4 (0.3)
	CHF in 30 days before surgery (%)		
	No	3600 (99.8)	1341 (99.6)
	Yes	9 (0.2)	6 (0.4)
	Hypertension requiring medication (%)		
	No	1651 (45.7)	616 (45.7)
	Yes	1958 (54.3)	731 (54.3)
	Dialysis before surgery (%)		
	No	3602 (99.8)	1345 (99.9)
	Yes	7 (0.2)	2 (0.1)
	Steroid use before surgery (%)		
	No	3526 (97.7)	1324 (98.3)
	Yes	83 (2.3)	23 (1.7)
	>10% weight loss (within 6 months) (%)		
	No	2973 (82.4)	1138 (84.5)
	Yes	636 (17.6)	209 (15.5)
	Bleeding disorders (%)		
	No	3513 (97.3)	1306 (97)
	Yes	96 (2.7)	41 (3)
	Transfusion before surgery (within 72 h) (%)		
	No	3586 (99.4)	1334 (99)
	Yes	23 (0.6)	13 (1)
	Systemic sepsis before surgery (%)		
	No	3571 (98.9)	1329 (98.7)
	Yes	38 (1.1)	18 (1.3)
	Preoperative serum sodium, mean (SD), mg/dl	138.7 (3.2)	139 (3.2)
	Preoperative BUN, mean (SD), mg/dl	15.1 (6.9)	15.7 (7.1)
	Preoperative serum creatinine, mean (SD), mg/dl	0.9 (0.4)	0.9 (0.4)
	Preoperative serum albumin, mean (SD), g/dl	3.8 (0.6)	3.8 (0.5)
	Preoperative total bilirubin, mean (SD), mg/dl	1.8 (2.6)	1.6 (2.3)
	Preoperative SGOT, mean (SD), U/l	50.1 (63.3)	49.6 (66.3)
	Preoperative alkaline phosphatase, mean (SD), U/l	186.3 (169.4)	175 (158.5)
	Preoperative WBC, mean (SD), 10^9^/l	7.4 (2.7)	7.4 (2.7)
	Preoperative hematocrit, mean (SD), vol%	37.6 (5.1)	37.6 (5)
	Preoperative platelet count, mean (SD), 10^9^/l	259.9 (93.3)	259 (93.3)
	Preoperative PTT, mean (SD), s	30.1 (4.9)	29.8 (5.5)
	Preoperative INR, mean (SD)	1 (0.2)	1.1 (0.3)
	Days from admission to operation, mean (SD), d	0.5 (2.1)	0.4 (1.9)
	Pancreatic duct size (%)		
	<3 mm	1034 (28.7)	416 (30.9)
	≥3 mm	2575 (71.3)	931 (69.1)
	ASA classification (%)		
	1	10 (0.3)	4 (0.3)
	2	800 (22.2)	278 (20.6)
	3	2618 (72.5)	989 (73.4)
	4	181 (5)	76 (5.6)
	Quarter of admission (%)		
	1	854 (23.7)	359 (26.7)
	2	953 (26.4)	338 (25.1)
	3	891 (24.7)	334 (24.8)
	4	911 (25.2)	316 (23.5)
	Preoperative obstructive jaundice (%)		
	No	1771 (49.1)	708 (52.6)
	Yes	1838 (50.9)	639 (47.4)
	Preoperative biliary stent (%)		
	No	1560 (43.2)	581 (43.1)
	Yes	2049 (56.8)	766 (56.9)
	Chemotherapy (within 90 days) (%)		
	No	2990 (82.8)	1080 (80.2)
	Yes	619 (17.2)	267 (19.8)
	Radiation therapy (within 90 days) (%)		
	No	3389 (93.9)	1255 (93.2)
	Yes	220 (6.1)	92 (6.8)
Intraoperative parameters	Total operation time, mean (SD), min	351.7 (127.9)	355.4 (131.7)
	Operative approach (%)		
	Open	3251 (90.1)	1214 (90.1)
	Laparoscopic	91 (2.5)	33 (2.4)
	Robotic	247 (6.8)	97 (7.2)
	Hybrid	20 (0.6)	3 (0.2)
	Pancreatic texture (%)		
	Soft	1606 (44.5)	597 (44.3)
	Intermediate/hard	2003 (55.5)	750 (55.7)
	Pancreatic reconstruction (%)		
	Pancreaticojejunal duct-to-mucosal	3248 (90)	1215 (90.2)
	Pancreaticogastrostomy	63 (1.7)	23 (1.7)
	Pancreaticojejunal invagination	298 (8.3)	109 (8.1)
	Vascular resection (%)		
	No	3127 (86.6)	1186 (88)
	Yes	482 (13.4)	161 (12)
Postoperative parameters	Number of bleeding transfusions, mean (SD)	0.2 (0.4)	0.2 (0.4)
	Number of SSI, mean (SD)	0.1 (0.4)	0.2 (0.4)
	Number of wound disruptions, mean (SD)	0 (0.1)	0 (0.1)
	Wound classification (%)		
	1	49 (1.4)	20 (1.5)
	2	2897 (80.3)	1072 (79.6)
	3	529 (14.7)	215 (16)
	4	134 (3.7)	40 (3)
	Wound infection (%)		
	No	3586 (99.4)	1338 (99.3)
	Yes	23 (0.6)	9 (0.7)
	Superficial incisional SSI (%)		
	No	3296 (91.3)	1243 (92.3)
	Yes	313 (8.7)	104 (7.7)
	Deep SSI (Deep incisional SSI/Organ or space SSI) (%)		
	No	3037 (84.2)	1107 (82.2)
	Yes	572 (15.8)	240 (17.8)
	Wound disruption (%)		
	No	3568 (98.9)	1335 (99.1)
	Yes	41 (1.1)	12 (0.9)
	Pneumonia after surgery (%)		
	No	3501 (97)	1303 (96.7)
	Yes	108 (3)	44 (3.3)
	Unplanned intubation (%)		
	No	3471 (96.2)	1296 (96.2)
	Yes	138 (3.8)	51 (3.8)
	Pulmonary embolism (%)		
	No	3564 (98.8)	1329 (98.7)
	Yes	45 (1.2)	18 (1.3)
	Ventilator >48 h (%)		
	No	3516 (97.4)	1308 (97.1)
	Yes	93 (2.6)	39 (2.9)
	Renal insufficiency/failure (%)		
	No	3550 (98.4)	1330 (98.7)
	Yes	59 (1.6)	17 (1.3)
	Urinary tract infection (%)		
	No	3510 (97.3)	1305 (96.9)
	Yes	99 (2.7)	42 (3.1)
	Stroke with neurological deficit (%)		
	No	3600 (99.8)	1343 (99.7)
	Yes	9 (0.2)	4 (0.3)
	Cardiac arrest (%)		
	No	3568 (98.9)	1333 (99)
	Yes	41 (1.1)	14 (1)
	Myocardial infarction (%)		
	No	3577 (99.1)	1328 (98.6)
	Yes	32 (0.9)	19 (1.4)
	Bleeding transfusion (%)		
	No	2989 (82.8)	1134 (84.2)
	Yes	620 (17.2)	213 (15.8)
	DVT (%)		
	No	3508 (97.2)	1304 (96.8)
	Yes	101 (2.8)	43 (3.2)
	Sepsis (%)		
	No	3286 (91.1)	1224 (90.9)
	Yes	323 (8.9)	123 (9.1)
	Septic shock (%)		
	No	3508 (97.2)	1304 (96.8)
	Yes	101 (2.8)	43 (3.2)
	Reoperation (%)		
	No	3429 (95)	1273 (94.5)
	Yes	180 (5)	74 (5.5)
	Wound closure (%)		
	All	3594 (99.6)	1344 (99.8)
	Not all	15 (0.4)	3 (0.2)
	Delayed gastric emptying (%)		
	No	3034 (84.1)	1140 (84.6)
	Yes	575 (15.9)	207 (15.4)
	Histology (%)		
	Pancreatic adenocarcinoma	2079 (57.6)	777 (57.7)
	Other	1530 (42.4)	570 (42.3)
	Pancreatic fistula (%)		
	No	3008 (83.3)	1123 (83.4)
	Grade B	568 (15.7)	211 (15.7)
	Grade C	33 (1)	13 (1)

CHF, congestive heart failure; COPD, chronic obstructive pulmonary disease; DVT, deep vein thrombosis; PD, pancreaticoduodenectomy; SSI, surgical site infection.

### Statistical analysis

All statistical analyses were conducted using R software (version 4.0.5). Quantitative variables are presented as mean (SD), while categorical variables are reported as count (%). In accordance with the latest risk assessment tool, Prediction Model Risk of Bias Assessment Tool (PROBAST), we avoided binary or multiclassification transformations of quantitative variables. This is because such conversions may lead to the loss of important information^25^, even though they may be nonlinearly related to the outcome variable. Similarly, univariate analysis was not implemented as it does not account for multivariate corrections, which may exclude important variables or identify spurious associations^25^. Lasso regression is a novel method for variable screening, which excludes the coefficients of relatively unimportant independent variables from the model by applying a penalized regression on all variable coefficients^26^. Thus, lasso regression can resolve the collinearity issue of variables, especially for screening high-dimensional variables^27^. In this study, we first screened the variables using lasso penalized regression, then used logistic regression to screen the variables and build a model, and finally developed a nomogram for CR-POPF based on the logistic model. We refer to this method as lasso-logistic regression for short. Furthermore, polynomial equations were extracted using the R package of nomogramEx. The optimal cutoff function and the Youden index were used to determine the thresholds for the two risk stratifications of the model^28^. Model similarity was evaluated using the likelihood ratio test, while model fitting was assessed using the Akaike information criterion (AIC) and Hosmer and Lemeshow tests. Model performance was evaluated using the area under the receiver operating characteristic curve (AUC) and misclassification error. The net benefit of the model was evaluated using the decision curve analysis (DCA) curve. The predictive power of the model was assessed using the clinical impact curve. A statistically significant difference was defined as *P*<0.05.

## Results

### Study cohort features

The training cohort comprised 3609 patients who underwent PD and were included in the ACS-NSQIP database from 2014 to 2017. The external validation cohort included 1,347 patients who underwent PD at NCC Hospital in China from 2014 to 2019. In the training cohort, there were 1958 men and 1651 women with a mean age of 64.8 years and a mean BMI of 27.4 kg/m². In the external validation cohort, there were 748 men and 599 women with a mean age of 65.6 years and a mean BMI of 27.4 kg/m². The incidence of CR-POPF was 16.7% (601/3609) in the training cohort and 16.6% (224/1347) in the validation cohort. The median times for ʻDays from operation until superficial incisional SSIʼ, ʻDays from operation until deep incisional SSIʼ, ʻDays from operation until organ/space incisional SSIʼ, and ʻDays from operation until highest amylase levelʼ were 10, 13, 12, and 3 days, respectively. The baseline characteristics of the two cohorts of patients who underwent PD are summarized in Table 1.

### Development of a novel prediction model

#### Lasso regression for variable screening

**Figure 2:**
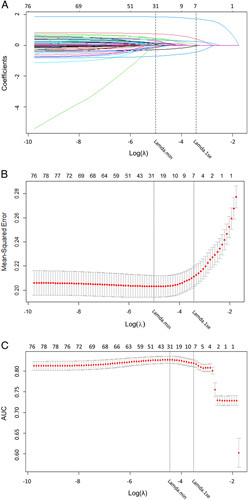
Lasso regression curves. (A) The curve of the regression coefficient versus log (λ); (B) the curve of MSE versus log (λ); (C) the curve of AUC versus log (λ). The λ.min represents the cutoff point at which MSE takes the minimum value, while λ.1se represents the point where MSE takes 1×standard error.

To mitigate the issue of multicollinearity and prevent overfitting of the model due to high-dimensional variables, we employed lasso regression to screen the initial 69 variables in the training cohort. The model variables were reduced to 31 when log(λ) reached the minimum mean squared error, and 7 when it reached a standard error (onefold SE) of the minimum distance, as determined by the lasso internal cross-validation (10 k-fold) (Fig. 2A, B). Although the difference in the AUC values of the models between the two cutoff points was not significant (Fig. 2C), we preferred to choose the point of onefold SE of the minimum distance (best λ value=0.024) as it was simpler and more explanatory. Lasso regression ultimately selected seven optimal variables (Table 2), including weight, pancreatic duct size, pancreatic texture, deep SSI, number of SSI, DGE, and pathology.

**Table 2:** Variables screened by Lasso penalized regression in the training cohort.

Best λ value	Variables in lasso model	Coefficient	OR
0.024	Weight	4.87E-05	1.00
	Pancreatic duct size	−2.75E-02	0.97
	Pancreatic texture	−4.36E-01	0.65
	Deep SSI	1.72E+00	5.59
	Number of SSI	2.46E-01	1.28
	DGE	4.14E-01	1.51
	Histology	6.22E-02	1.06

DGE, delayed gastric emptying; SSI, surgical site infection.

#### Development of a predictive nomogram based on logistic regression, extraction of scoring equation, and risk stratification

**Figure 3:**
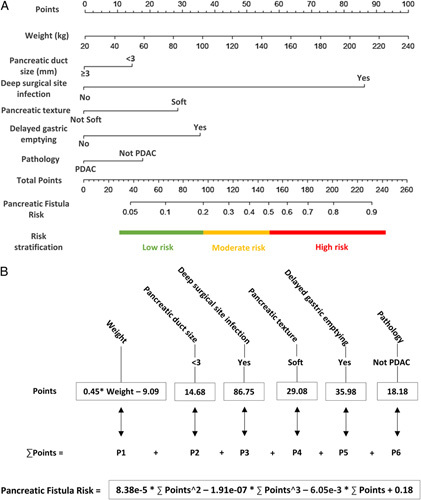
The nomogram for predicting POPF based on the lasso-logistic regression. (A) Nomogram representing the point assignment for each variable to calculate the predicted probability of POPF, the corresponding three risk stratifications are labeled at the bottom; (B) Risk calculation formula: The value of each variable corresponds to different points, and the risk value can be obtained by summing the points and inputting them into the risk formula.

Next, we incorporated the seven variables screened by lasso regression into logistic regression for further modeling. However, the variable ʻnumber of SSIʼ was excluded from the model as it was not an independent risk factor for CR-POPF (*P*>0.05) (Supplementary Table 1, Supplemental Digital Content 2, http://links.lww.com/JS9/A952). This exclusion was likely because the indicator was associated with the presence of deep SSI. Therefore, we reincorporated the remaining six variables into logistic regression, and the results indicated that they were all independent risk factors for CR-POPF (*P*<0.001). There were no collinearity issues among the variables (VIF<2) (Table 3; Supplementary Figure 1, Supplemental Digital Content 3, http://links.lww.com/JS9/A953), indicating successful modeling. We developed a nomogram for predicting the incidence of CR-POPF based on the logistic regression model (Fig. 3A). The nomogram includes six predictive indicators, comprising two preoperative indicators (weight and pancreatic duct size), one intraoperative indicator (pancreatic texture), and three postoperative indicators (deep SSI, DGE, and pathology). However, due to limitations on the scale of the axis in the nomogram, we were unable to obtain the exact probability for the quantitative indicators, which limits its clinical applicability. To address this issue, we extracted polynomial equations for the predictors to calculate the exact score for each indicator and the corresponding DGE risk associated with the total score (Fig. 3B). The risk of PF was calculated using the following formula:


$$\mathrm{PF Risk}=8.38\times 10^{-5}\times {\mathrm{(\sum Points)}}^{2}-1.91\times {\mathrm{10}}^{\mathrm{-7}}\;\times {\mathrm{(\sum Points)}}^{3}-6.05 \times {\mathrm{10}}^{\mathrm{-3}}\times (\sum Points) + 0.18$$

**Table 3:** Independent variables included in the nomogram based on the lasso-logistic regression in the training cohort.

Nomogram model	VIF	OR (95% CI)	*P*
Weight	1.01	1.01 (1.01–1.02)	<0.001
Pancreatic duct size	1.11	0.68 (0.54–0.85)	<0.001
Pancreatic gland texture	1.16	0.46 (0.37–0.58)	<0.001
Deep SSI	1.01	9.96 (8.01–12.41)	<0.001
DGE	1.01	2.59 (2.04–3.30)	<0.001
Histology	1.08	1.62 (1.31–2.01)	<0.001

VIF less than 2, there is no collinearity between independent variables.

DGE, delayed gastric emptying; SSI, surgical site infection; VIF, variance inflation factor.

Where ∑ Points represents the sum of points based on relevant clinical factors.

To further stratify the risk of POPF, we applied the optimal cutoff function and the Youden index to calculate the binary risk stratification threshold of the prediction model in the training cohort. We obtained two different thresholds, 0.5 and 0.2, which we named optimal cutoff value and best cutoff value, respectively (Supplementary Figure 2A, Supplemental Digital Content 3, http://links.lww.com/JS9/A953). We then used the intersection of the two thresholds to create three risk stratifications: low-risk, intermediate-risk, and high-risk groups. These corresponded to predicted probabilities of less than or equal to 0.2, greater than 0.2 and less than or equal to 0.5, and greater than 0.5, respectively. We evaluated the discriminative ability of this risk stratification method by presenting the receiver operating characteristic curves of the models within each risk group in the training and validation cohorts (Supplementary Figure 2B, Supplemental Digital Content 3, http://links.lww.com/JS9/A953). The results showed that all three risk groups had good discriminative ability, particularly the low-risk and high-risk groups. We further analyzed the actual incidence of PF in each stratification group and found that this three-stratification method had good stratification performance in both the training and validation cohorts (Supplementary Figure 2C, Supplemental Digital Content 3, http://links.lww.com/JS9/A953, Supplementary Table 2, Supplemental Digital Content 4, http://links.lww.com/JS9/A954).

### Evaluation and external validation of the novel model

#### Advantages of Lasso regression for screening modeling variables

**Figure 4:**
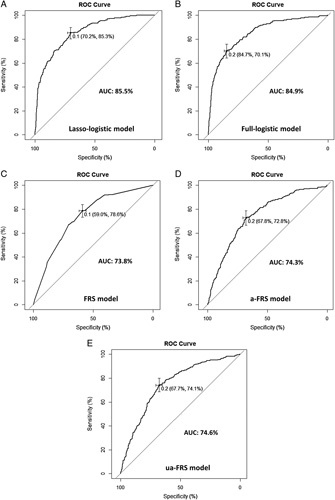
ROC curves of five different models in the validation cohort. (A) lasso-logistic model; (B) Full-logistic model; (C) FRS model; (D) a-FRS model; (E) ua-FRS model. The figure marks the best cutoff value, specificity, sensitivity, and area under the ROC curve (AUC) value for each model.

We introduced a novel modeling method called the lasso-logistic model, which involves preliminary variable screening using lasso penalized regression before incorporating variables into logistic regression for modeling. In contrast, conventional methods of variable screening include univariate analysis and full variable incorporation. However, univariate analysis is not recommended in the latest PROBAST guideline. To compare the performance of the lasso-logistic model with the full-logistic model, which includes all variables, we conducted a comparative analysis. The results showed that the two prediction models were statistically different (*P*=0.006, likelihood ratio test) (Table 4). The lasso-logistic model had fewer independent variables (6 vs. 13), better model fit (AIC, 2435.5 vs. 2472.7), and higher model discrimination in the validation cohort (AUC, 85.5 vs. 84.9%) (Table 4; Fig. 4A, B). Importantly, there was no significant increase in misclassification error (12.8 vs. 12.6%) (Table 4). These findings suggest that lasso regression is a better variable screening method than full variable inclusion. In conclusion, the lasso-logistic model outperformed the full-logistic model.

**Table 4:** Evaluation and comparison of two variable screening methods in the validation cohort.

Models	Variable inclusion methods	Independent variables number (*P*<0.05)	AIC	Misclass error (%)	AUC (%)	Likelihood ratio test (*P*)
Lasso-logistic model	Lasso regression	6	2435.5	12.8	85.5	0.006
Full-logistic model	Full variables	13	2472.7	12.6	84.9	

AIC is a standard measure of the goodness of model fit. A lower AIC value indicates a better model fit.

AIC, Akaike information criterion; AUC, area under ROC curve.

#### Advantages of the lasso-logistic model compared to conventional models

**Figure 5:**
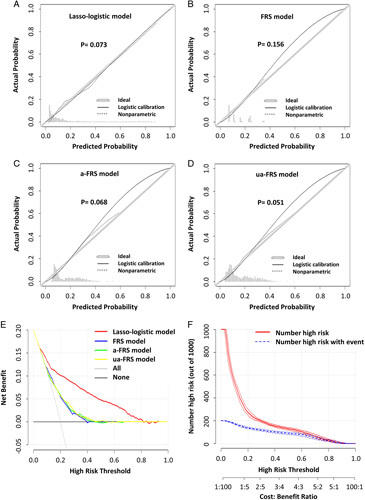
Evaluation of different models in the validation cohort. (A–D) Calibration curves of four different models: (A) Lasso-logistic model; (B) FRS model; (C) a-FRS model; (D) ua-FRS model. The *P*-value is obtained from the Hosmer and Lemeshow goodness of fit (GOF) test. A *P*-value >0.05 indicates a good model fit. (E) Decision curve analysis (DCA) of four different models. (F) Clinical impact curve of the lasso-logistic model. The red curve represents the number of predicted cases of POPF using the lasso-logistic model under the threshold probability, while the blue curve represents the actual number of cases of POPF that occurred under the threshold probability.

Although three previously reported CR-POPF risk scoring systems—FRS, a-FRS, and ua-FRS—differ from the new model in terms of accurate risk prediction, they are all created based on logistic regression, as is the new model. Therefore, to facilitate comparison, we evaluated and validated these four logistic models. The analysis results (Table 5) showed that the three conventional models (FRS model, a-FRS model, and ua-FRS model) were all statistically different from the lasso-logistic model (*P*<0.001, likelihood ratio test). Compared to the three conventional models, the lasso-logistic model had a better fit (AIC, 2435.5 vs. 3039.2, 3036.2, 3019.2) (Fig. 5A, B, C, D), higher model discrimination in the validation cohort (AUC, 85.5 vs. 73.8%, 74.3, 74.6%) (Fig. 4A, C, D, E), and minimal misclassification error (12.8 vs. 16.6%, 16.7, 16.6%). Furthermore, the new model increased only a limited number of independent variables (6 vs. 4, 3, and 4). The DCA curve (Fig. 5E) showed that the lasso-logistic model had a significantly higher net benefit in the threshold probability range of 0.05–0.82 than the other three conventional models. Figure 5F illustrated the relationship between the number of CR-POPF cases predicted by the lasso-logistic model and the true positive number under different threshold probabilities (with a base set at 1000). Therefore, the lasso-logistic model was superior to the three conventional models and its slight increase in the number of independent variables did not significantly affect the ease of use or clinical applicability.

**Table 5:** Evaluation and comparison of four different models in the validation cohort.

Models	Independent variables number (*P*<0.05)	AIC	Misclass error (%)	AUC (%)	Hosmer and Lemeshow test (*P*)	Likelihood ratio test (*P*)
Lasso-logistic model	6	2435.5	12.8	85.5	0.073	1^a^
FRS model	4	3039.2	16.6	73.8	0.156	<0.001
a-FRS model	3	3036.2	16.7	74.3	0.068	<0.001
ua-FRS model	4	3019.2	16.6	74.6	0.051	<0.001

a Compared with lasso-logistic model. AIC is a standard measure of the goodness of model fit. A lower AIC value indicates a better model fit.

AIC, Akaike information criterion; AUC, area under ROC curve.

## Discussion

Effective treatment of CR-POPF remains a significant challenge in pancreatic surgery^29^. Accurate prediction models for CR-POPF development can help in providing proactive strategies for preventive management and early intervention. In this study, we included a total of 69 indicators that may affect CR-POPF preoperatively, intraoperatively, and postoperatively, and utilized a reliable lasso-logistic method for modeling. We identified six independent risk factors—weight, pancreatic duct size, pancreatic texture, deep SSI, DGE, and pathology—that affect CR-POPF. Finally, we established a nomogram for predicting the risk of CR-POPF, extracted the calculation formula, and stratified the risk. This model allows for sustained, dynamic, and accurate CR-POPF risk prediction in PD patients and has broad clinical applications due to the low number of model variables.

In 2013, Callery *et al*.^5^ proposed an FRS system based on preoperative and intraoperative indicators. The original FRS was a logistic regression model based on four indicators: pancreatic texture, duct size, blood loss, and pathology. An a-FRS system was proposed by Mungroup *et al*.^6^ in 2017. The a-FRS consists of three indicators: pancreatic texture, pancreatic duct diameter, and BMI. In 2019, Mungroup *et al*.^7^ further developed an ua-FRS system. The ua-FRS contains four indicators of pancreatic texture, duct size, BMI, and sex. The three independent variables of pancreatic duct size, pancreatic texture, and pathology were also included in this new model, indicating their significant value in CR-POPF prediction. A small pancreatic duct diameter and soft pancreatic tissue are recognized as the most common risk factors for POPF, as smaller ducts may not be suitable for suturing, especially using the pancreatic duct-to-mucosa anastomosis approach^5^. Similarly, soft pancreas tissue is prone to tissue tearing and leakage during surgical anastomosis^30^. In addition, a small pancreatic duct and soft pancreas usually indicate good exocrine function, and the secretion of pancreatic fluid, rich in proteolytic enzymes, increases the chance of anastomotic leakage. In contrast, PDAC has a lower incidence of POPF than other pathological types due to stromal hyperplasia and inflammatory infiltration, which harden pancreatic tissue and inhibit pancreatic exocrine function^5^. Interestingly, this study showed that body weight, rather than BMI, was independently associated with CR-POPF occurrence, suggesting that weight is a more significant indicator, while height as a secondary indicator may dilute the correlation of BMI. Compared to BMI, a weight-standardized index, the distribution of fat in the abdominal viscera is more likely to affect fatty infiltration of pancreatic tissue, alter texture, and exocrine function^31–33^, and be associated with the difficulty of PD reconstruction^30^. Thus, this provides a possible explanation for the more significant predictive value of body weight in this study. Additionally, excluding the effect of height also increases the convenience of data acquisition in the new model.

It is worth mentioning that the new model introduced two important postoperative indicators, deep SSI and DGE, which significantly increased the AUC value of the external validation cohort to 85.5%, higher than the previous three conventional models^5–7^. Infection is one of the most common postoperative complications, with an incidence of SSI after PD ranging from 17 to 28%^34,35^. SSI are strongly associated with POPF, but their causality remains controversial^13,36^. In clinical practice, early identification of POPF may be challenging, especially in centers that implement Enhanced Recovery After Surgery (ERAS) protocols, which encourage early removal or even avoidance of abdominal drainage. DGE occurs in up to 40–60% of cases after PD^37–39^, and the pathophysiological mechanisms underlying its development remain incompletely understood and may be related to hemorrhage, edema, or neurological trauma due to surgery^40^. Similar to SSI, the causality between DGE and POPF is currently debated, despite their strong association^15,41,42^. Our study confirmed the correlation between deep SSI, DGE, and CR-POPF. More importantly, we were able to use this correlation to predict CR-POPF before its diagnosis is confirmed, regardless of the causal relationship. Therefore, deep SSI and DGE may serve as valuable predictors of CR-POPF in clinical practice.

The study has several limitations. First, pancreatic gland texture was measured subjectively by the operating surgeon and classified into soft and nonsoft, rather than based on an objective criterion. Second, the new model was developed and evaluated in separate national cohorts and may not be generally representative across surgeons in terms of technical approach and postoperative management. It is unclear whether similar results could be obtained in other surgical centers with different perioperative management strategies. Finally, the study was retrospective and excluded missing data. However, the distribution of missing data may not be random, which makes it impossible to exclude residual confounding. Additionally, some data related to the timing sequence of variables were missing, which may have affected the predictive ability of the model. Despite the better performance of the nomogram in predicting POPF, it still has some shortcomings that need to be addressed, such as (1) its inability to predict PF early, as postoperative indicators are required in the model, and (2) the complexity of diagnosing SSI and DGE, which may affect the usability of the model. Therefore, further validation of our findings is necessary through prospective, multicenter clinical studies. Nonetheless, this study is unique in using a large volume dataset and a large number of variables from the multicenter ACS-NSQIP database across the United States for modeling and validation in an international cohort. Importantly, we utilized a reliable statistical method to screen high-dimensional variables and constructed a new prediction model. We excluded all postoperative indicators used to diagnose CR-POPF, such as amylase values of postoperative drainage fluid and peritoneal fluid drainage, as they would have amplified the predictive power of the model. Furthermore, the new model added only two variables with significantly higher AUC values compared to the recent ua-FRS model. This has made the new model simple and accessible for clinical use.

## Conclusion

In this study, we utilized a reliable lasso-logistic statistical method to develop a simple nomogram for predicting CR-POPF in PD patients. Our model achieved sustained, dynamic, and precise CR-POPF prediction with a limited number of variables and easy clinical application. By proactively predicting, identifying, and managing PFs, our new model will enable surgeons to obtain better outcomes from this daunting postoperative complication.

## Ethical approval

The China cohort was approved for this study by the Ethics Committee of Chinese NCC (No. 17-168/1424).

## Sources of funding

1. National Natural Science Foundation of China (No. 81972314, 81802463).

2. CAMS Innovation Fund for Medical Sciences (No. 2022-I2M-1-010).

## Author contribution

Z.G.: conceptualization, data curation, formal analysis, methodology, software, visualization, writing – original draft; Y.D.: conceptualization, data curation, methodology, software, visualization, writing – original draft; P.W.: conceptualization, data curation, methodology, visualization, writing – original draft; X.Z.: formal analysis, methodology, software, visualization; J.H.: data curation, supervision, writing – review and editing; C.W.: conceptualization, supervision, writing – review and editing; J.Z.: conceptualization, data curation, methodology, supervision, writing – review and editing.

## Conflicts of interest disclosure

All other authors declare that they have no competing interests.

## Research registration unique identifying number (UIN)

1. Name of the registry: development and validation of a nomogram to predict postoperative pancreatic fistula after pancreatoduodenectomy: a multi-institutional observational study.
2. Unique Identifying number or registration ID: researchregistry8530.
3. Hyperlink to your specific registration (must be publicly accessible and will be checked): https://www.researchregistry.com/browse-theregistry#home/registrationdetails/639003bcf2522b0021446f1d/.

## Guarantor

Jianwei Zhang, Department of Pancreatic and Gastric Surgery, National Cancer Center/Cancer Hospital, Chinese Academy of Medical Sciences and Peking Union Medical College, Beijing, People’s Republic of China. E-mail: academicpaper@hotmail.com.

## Data availability statement

The datasets generated during and/or analyzed during the current study are available in the National Surgical Quality Improvement Program (NSQIP) repository, https://www.facs.org/quality-programs/acs-nsqip/participant-use. Further inquiries can be directed to the corresponding author.

## Provenance and peer review

Not commissioned, externally peer reviewed.

## Supplementary Material

SUPPLEMENTARY MATERIAL

## References

[1] Del Chiaro M Valente R Arnelo U. Minimally invasive pancreaticoduodenectomy for the treatment of pancreatic-head and periampullary tumors. JAMA Surg 2017;152:343. 28030664 10.1001/jamasurg.2016.4754

[2] Griffin JF Poruk KE Wolfgang CL. Pancreatic cancer surgery: past, present, and future. Chin J Cancer Res 2015;27:332–348. 26361403 10.3978/j.issn.1000-9604.2015.06.07 PMC4560737

[3] Bannone E Andrianello S Marchegiani G. Postoperative hyperamylasemia (POH) and acute pancreatitis after pancreatoduodenectomy (POAP): state of the art and systematic review. Surgery 2021;169:377–87. 32641279 10.1016/j.surg.2020.04.062

[4] Andrianello S Marchegiani G Malleo G. Pancreaticojejunostomy with externalized stent vs pancreaticogastrostomy with externalized stent for patients with high-risk pancreatic anastomosis: a single-center, phase 3, randomized clinical trial. JAMA Surg 2020;155:313–321. 32101272 10.1001/jamasurg.2019.6035 PMC7160692

[5] Callery MP Pratt WB Kent TS. A prospectively validated clinical risk score accurately predicts pancreatic fistula after pancreatoduodenectomy. J Am Coll Surg 2013;216:1–14. 23122535 10.1016/j.jamcollsurg.2012.09.002

[6] Mungroop TH van Rijssen LB van Klaveren D. Alternative fistula risk score for pancreatoduodenectomy (a-frs): design and international external validation. Ann Surg 2019;269:937–43. 29240007 10.1097/SLA.0000000000002620

[7] Mungroop TH Klompmaker S Wellner UF. Updated alternative fistula risk score (ua-frs) to include minimally invasive pancreatoduodenectomy: pan-european validation. Ann Surg 2021;273:334–40. 30829699 10.1097/SLA.0000000000003234

[8] Shinde RS Acharya R Chaudhari VA. External validation and comparison of the original, alternative and updated-alternative fistula risk scores for the prediction of postoperative pancreatic fistula after pancreatoduodenectomy. Pancreatology 2020;20:751–756. 32340876 10.1016/j.pan.2020.04.006

[9] Group PS Writing C Pande R. External validation of postoperative pancreatic fistula prediction scores in pancreatoduodenectomy: a systematic review and meta-analysis. HPB (Oxford) 2022;24:287–98. 34810093 10.1016/j.hpb.2021.10.006

[10] Li B Pu N Chen Q. Comprehensive diagnostic nomogram for predicting clinically relevant postoperative pancreatic fistula after pancreatoduodenectomy. Front Oncol 2021;11:717087. 34277458 10.3389/fonc.2021.717087 PMC8281206

[11] Huang XT Huang CS Liu C. Development and validation of a new nomogram for predicting clinically relevant postoperative pancreatic fistula after pancreatoduodenectomy. World J Surg 2021;45:261–269. 32901325 10.1007/s00268-020-05773-y

[12] Shen J Guo F Sun Y. Predictive nomogram for postoperative pancreatic fistula following pancreaticoduodenectomy: a retrospective study. BMC Cancer 2021;21:550. 33992090 10.1186/s12885-021-08201-z PMC8126152

[13] Parikh JA Beane JD Kilbane EM. Is American College of Surgeons NSQIP organ space infection a surrogate for pancreatic fistula? J Am Coll Surg 2014;219:1111–1116. 25442065 10.1016/j.jamcollsurg.2014.08.006 PMC4386682

[14] Nanashima A Abo T Arai J. Clinicopathological parameters associated with surgical site infections in patients who underwent pancreatic resection. Hepatogastroenterology 2014;61:1739–1743. 25436372

[15] Robinson JR Marincola P Shelton J. Peri-operative risk factors for delayed gastric emptying after a pancreaticoduodenectomy. HPB (Oxford) 2015;17:495–501. 25728447 10.1111/hpb.12385 PMC4430779

[16] Pitt HA Kilbane M Strasberg SM. ACS-NSQIP has the potential to create an HPB-NSQIP option. HPB (Oxford) 2009;11:405–413. 19768145 10.1111/j.1477-2574.2009.00074.x PMC2742610

[17] Shiloach M Frencher SK Jr Steeger JE. Toward robust information: data quality and inter-rater reliability in the American College of Surgeons National Surgical Quality Improvement Program. J Am Coll Surg 2010;210:6–16. 20123325 10.1016/j.jamcollsurg.2009.09.031

[18] Collins GS Reitsma JB Altman DG. Transparent Reporting of a multivariable prediction model for Individual Prognosis or Diagnosis (TRIPOD): the TRIPOD statement. Ann Intern Med 2015;162:55–63. 25560714 10.7326/M14-0697

[19] Mathew G Agha R Group S. STROCSS 2021: strengthening the reporting of cohort, cross-sectional and case–control studies in surgery. Int J Surg 2021;96:106165. 34774726 10.1016/j.ijsu.2021.106165

[20] Bassi C Marchegiani G Dervenis C. The 2016 update of the International Study Group (ISGPS) definition and grading of postoperative pancreatic fistula: 11 years after. Surgery 2017;161:584–591. 28040257 10.1016/j.surg.2016.11.014

[21] Wente MN Bassi C Dervenis C. Delayed gastric emptying (DGE) after pancreatic surgery: a suggested definition by the International Study Group of Pancreatic Surgery (ISGPS). Surgery 2007;142:761–768. 17981197 10.1016/j.surg.2007.05.005

[22] Pulvirenti A Ramera M Bassi C. Modifications in the International Study Group for Pancreatic Surgery (ISGPS) definition of postoperative pancreatic fistula. Transl Gastroenterol Hepatol 2017;2:107. 29354764 10.21037/tgh.2017.11.14 PMC5763010

[23] Mangram AJ Horan TC Pearson ML. Guideline for Prevention of Surgical Site Infection, 1999. Centers for Disease Control and Prevention (CDC) Hospital Infection Control Practices Advisory Committee. Am J Infect Control 1999;27:97–132; quiz 3-4; discussion 96. 10196487

[24] Hadidi A. Pancreatic duct diameter: sonographic measurement in normal subjects. J Clin Ultrasound 1983;11:17–22. 6403583 10.1002/jcu.1870110105

[25] Wolff RF Moons KGM Riley RD. PROBAST: a tool to assess the risk of bias and applicability of prediction model studies. Ann Intern Med 2019;170:51–58. 30596875 10.7326/M18-1376

[26] Tibshirani R. The lasso method for variable selection in the Cox model. Stat Med 1997;16:385–395. 9044528 10.1002/(sici)1097-0258(19970228)16:4<385::aid-sim380>3.0.co;2-3

[27] Bunea F She Y Ombao H. Penalized least squares regression methods and applications to neuroimaging. Neuroimage 2011;55:1519–1527. 21167288 10.1016/j.neuroimage.2010.12.028 PMC5485905

[28] Unal I. Defining an optimal cut-point value in ROC analysis: an alternative approach. Comput Math Methods Med 2017;2017:3762651–14. 28642804 10.1155/2017/3762651 PMC5470053

[29] Bassi C Marchegiani G Giuliani T. Pancreatoduodenectomy at the Verona Pancreas Institute: the evolution of indications, surgical techniques and outcomes: a retrospective analysis of 3000 consecutive cases. Ann Surg 2021;276:1029–1038. 33630454 10.1097/SLA.0000000000004753

[30] Mathur A Pitt HA Marine M. Fatty pancreas: a factor in postoperative pancreatic fistula. Ann Surg 2007;246:1058–1064. 18043111 10.1097/SLA.0b013e31814a6906

[31] Gaujoux S Cortes A Couvelard A. Fatty pancreas and increased body mass index are risk factors of pancreatic fistula after pancreaticoduodenectomy. Surgery 2010;148:15–23. 20138325 10.1016/j.surg.2009.12.005

[32] Pecorelli N Carrara G De Cobelli F. Effect of sarcopenia and visceral obesity on mortality and pancreatic fistula following pancreatic cancer surgery. Br J Surg 2016;103:434–442. 26780231 10.1002/bjs.10063

[33] Zhou L Xiao WM Li CP. Impact of fatty pancreas on postoperative pancreatic fistulae: a meta-analysis. Front Oncol 2021;11:622282. 34926236 10.3389/fonc.2021.622282 PMC8671996

[34] Poruk KE Lin JA Cooper MA. A novel, validated risk score to predict surgical site infection after pancreaticoduodenectomy. HPB (Oxford) 2016;18:893–899. 27624516 10.1016/j.hpb.2016.07.011 PMC5094482

[35] Morikane K. Epidemiology and risk factors associated with surgical site infection after different types of hepatobiliary and pancreatic surgery. Surg Today 2017;47:1208–14. 28303341 10.1007/s00595-017-1503-0

[36] Suragul W Rungsakulkij N Vassanasiri W. Predictors of surgical site infection after pancreaticoduodenectomy. BMC Gastroenterol 2020;20:201. 32586351 10.1186/s12876-020-01350-8 PMC7318744

[37] Ellis RJ Gupta AR Hewitt DB. Risk factors for post-pancreaticoduodenectomy delayed gastric emptying in the absence of pancreatic fistula or intra-abdominal infection. J Surg Oncol 2019;119:925–31. 30737792 10.1002/jso.25398 PMC7747058

[38] Haltmeier T Kaderli RM Kurmann A. Delayed gastric emptying after pancreaticoduodenectomy: analysis of associated risk factors. Am Surg 2015;81:E392–E394. 26672577

[39] Noorani A Rangelova E Del Chiaro M. Delayed gastric emptying after pancreatic surgery: analysis of factors determinant for the short-term outcome. Front Surg 2016;3:25. 27200357 10.3389/fsurg.2016.00025 PMC4843166

[40] Nishio RT Pacheco-Jr AM Moricz A. What factors contribute to delayed gastric emptying after duodenopancreatectomy with piloric preservation? Arq Bras Cir Dig 2021;34:e1592. 34669882 10.1590/0102-672020210002e1592 PMC8521836

[41] Parmar AD Sheffield KM Vargas GM. Factors associated with delayed gastric emptying after pancreaticoduodenectomy. HPB (Oxford) 2013;15:763–772. 23869542 10.1111/hpb.12129 PMC3791115

[42] Zhou Y Lin L Wu L. A case-matched comparison and meta-analysis comparing pylorus-resecting pancreaticoduodenectomy with pylorus-preserving pancreaticoduodenectomy for the incidence of postoperative delayed gastric emptying. HPB (Oxford) 2015;17:337–343. 25388024 10.1111/hpb.12358 PMC4368398

