# Supplementary material for: Development and validation of a novel nomogram to predict postoperative pancreatic fistula after pancreatoduodenectomy using lasso-logistic regression: an international multi-institutional observational study
Source: Int J Surg. 2023 Sep 5;109(12):4027–40. doi: 10.1097/JS9.0000000000000695 (PMC10720876; doi:10.1097/JS9.0000000000000695)
Supplement: SUPPLEMENTARY MATERIAL [file js9-109-4027-s002.docx]

| Lasso-logistic model | VIF | OR | 95% CI | P value |
| --- | --- | --- | --- | --- |
| Weight | 1.01 | 1.01 | 1.01-1.02 | <0.001 |
| Pancreatic duct size | 1.11 | 0.68 | 0.54-0.85 | <0.001 |
| Pancreatic gland texture | 1.16 | 0.46 | 0.37-0.58 | <0.001 |
| Deep SSI | **5.55** | 6.89 | 4.09-11.50 | <0.001 |
| Number of SSI | **5.54** | 1.49 | 0.90-2.51 | **0.122** |
| DGE | 1.01 | 2.59 | 2.04-3.29 | <0.001 |
| Histology | 1.08 | 1.61 | 1.30-2.00 | <0.001 |

*P* < 0.05, variables are independent in the model. Abbreviations: SSI, surgical site infection; DGE, delayed gastric emptying; VIF, variance inflation factor.
