# Supplementary material for: Development and validation of a novel nomogram to predict postoperative pancreatic fistula after pancreatoduodenectomy using lasso-logistic regression: an international multi-institutional observational study
Source: Int J Surg. 2023 Sep 5;109(12):4027–40. doi: 10.1097/JS9.0000000000000695 (PMC10720876; doi:10.1097/JS9.0000000000000695)
Supplement: SUPPLEMENTARY MATERIAL [file js9-109-4027-s003.docx]

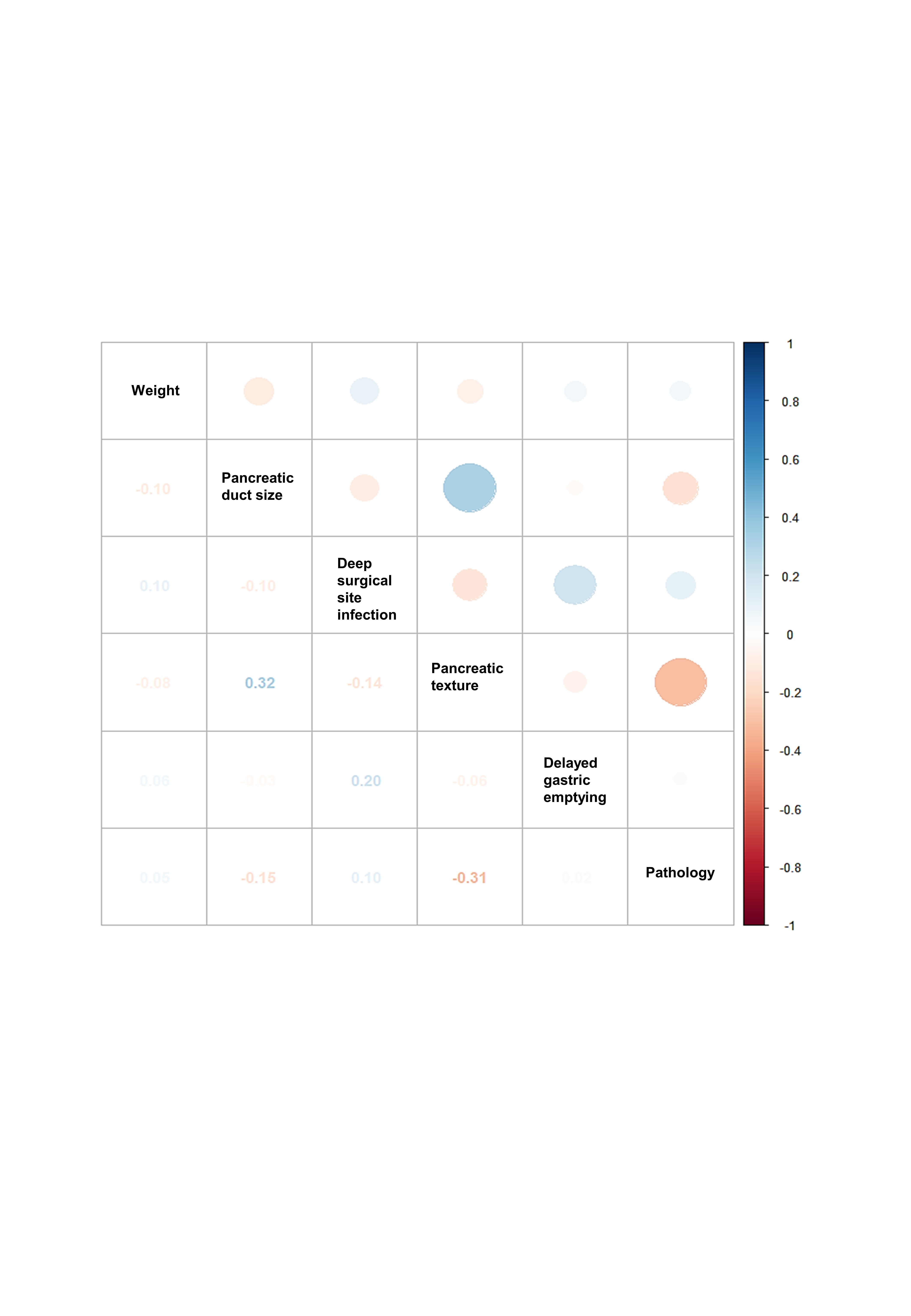


**Supplementary Figure 1** Heatmap of correlations between variables in the Lasso-logistic model. The heatmap displays the correlations between variables in the Lasso-logistic model. No significant correlations are observed among the variables.


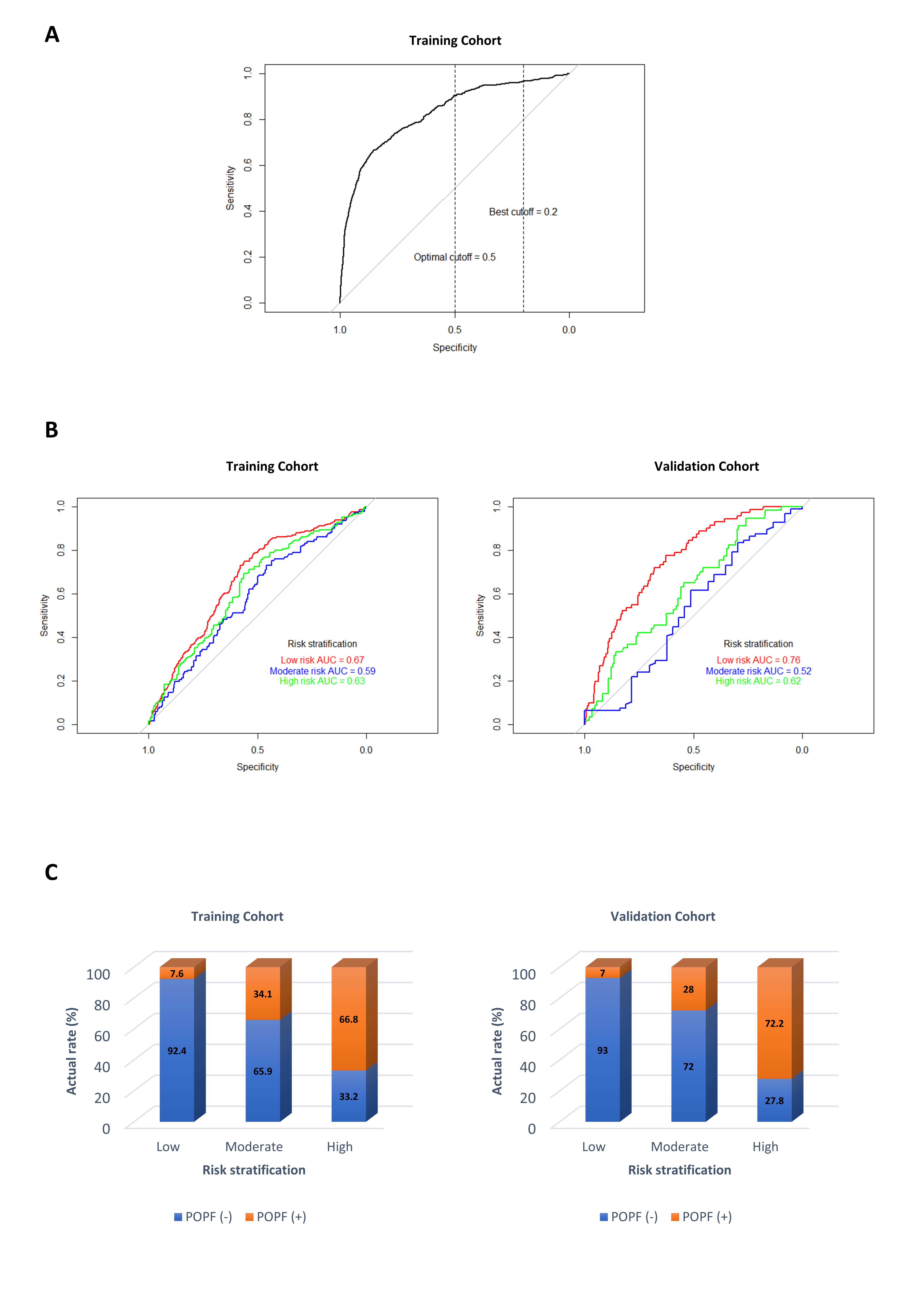


**Supplementary Figure 2** Establishment and evaluation of the risk stratification model. (**A**) Two thresholds for risk stratification; (**B**) ROC curves of the models within each stratification group in the training and validation cohorts; (**C**) Actual incidence of pancreatic fistula in each risk stratification group.
