# Supplementary material for: Development and validation of a novel nomogram to predict postoperative pancreatic fistula after pancreatoduodenectomy using lasso-logistic regression: an international multi-institutional observational study
Source: Int J Surg. 2023 Sep 5;109(12):4027–40. doi: 10.1097/JS9.0000000000000695 (PMC10720876; doi:10.1097/JS9.0000000000000695)
Supplement: SUPPLEMENTARY MATERIAL [file js9-109-4027-s004.docx]

|  | Training cohort (n=3069) | | | | | | Validation cohort (n=1347) | | | | | |
| --- | --- | --- | --- | --- | --- | --- | --- | --- | --- | --- | --- | --- |
| Risk group | N | POPF (+) | | POPF (-) | Positive rate (%) | Negative rate (%) | N | POPF (+) | POPF (-) | Positive rate (%) | Negative rate (%) |  |
| Low risk | 2833 | | 214 | 2619 | 7.6 | 92.4 | 1010 | 71 | 939 | 7 | 93 |  |
| Moderate risk | 402 | | 137 | 265 | 34.1 | 65.9 | 204 | 57 | 147 | 28 | 72 |  |
| High risk | 374 | | 250 | 124 | 66.8 | 33.2 | 133 | 96 | 37 | 72.2 | 27.8 |  |
